# Protective Effect of Antioxidants on Neuronal Dysfunction and Plasticity in Huntington's Disease

**DOI:** 10.1155/2017/3279061

**Published:** 2017-01-12

**Authors:** Thirunavukkarasu Velusamy, Archana S. Panneerselvam, Meera Purushottam, Muthuswamy Anusuyadevi, Pramod Kumar Pal, Sanjeev Jain, Musthafa Mohamed Essa, Gilles J. Guillemin, Mahesh Kandasamy

**Affiliations:** ^1^Department of Biotechnology, Bharathiar University, Coimbatore, Tamil Nadu, India; ^2^DBT Ramalingaswami Re-Entry Fellowship Programme, Department of Biotechnology (DBT), New Delhi, India; ^3^Laboratory of Stem Cells and Neuroregeneration, Department of Animal Science, School of Life Sciences, Bharathidasan University, Tiruchirappalli, Tamil Nadu, India; ^4^Department of Psychiatry, National Institute of Mental Health and Neurosciences (NIMHANS), Bangalore, Karnataka, India; ^5^Molecular Gerontology Laboratory, Department of Biochemistry, School of Life Sciences, Bharathidasan University, Tiruchirappalli, Tamil Nadu, India; ^6^Department of Neurology, National Institute of Mental Health and Neurosciences (NIMHANS), Bangalore, Karnataka, India; ^7^Department of Food Science and Nutrition, CAMS, Sultan Qaboos University, Muscat, Oman; ^8^Neuroinflammation Group, Faculty of Medicine and Health Sciences, Macquarie University, Sydney, NSW, Australia; ^9^UGC-Faculty Recharge Program (UGC-FRP), University Grant Commission, New Delhi, India

## Abstract

Huntington's disease (HD) is characterised by movement disorders, cognitive impairments, and psychiatric problems. The abnormal generation of reactive oxygen species and the resulting oxidative stress-induced mitochondrial damage in neurons upon CAG mutations in the* HTT* gene have been hypothesized as the contributing factors of neurodegeneration in HD. The potential use of antioxidants against free radical toxicity has been an emerging field in the management of ageing and many neurodegenerative disorders. Neural stem cells derived adult neurogenesis represents the regenerative capacity of the adult brain. The process of adult neurogenesis has been implicated in the cognitive functions of the brain and is highly modulated positively by different factors including antioxidants. The supportive role of antioxidants to reduce the severity of HD via promoting the functional neurogenesis and neuroprotection in the pathological adult brain has great promise. This review comprehends the recent studies describing the therapeutic roles of antioxidants in HD and other neurologic disorders and highlights the scope of using antioxidants to promote adult neurogenesis in HD. It also advocates a new line of research to delineate the mechanisms by which antioxidants promote adult neurogenesis in HD.

## 1. Introduction

Huntington's disease (HD) is an autosomal dominant neurodegenerative syndrome associated with abnormal CAG expansions in the Huntington* (HTT)* gene [1–3]. The mutant* HTT* contains polymorphic CAG repeats in excess of 39 in exon 1 of the gene present in the short arm of the chromosome 4 [3–5]. The CAG mutations ultimately result in the abnormal expansion of polyglutamine (polyQ) tracts in the HTT protein, which leads to misfolding and loss of protein function [6, 7]. The polyQ expansion has been identified to be the primary inducer of degeneration of medium spiny neurons (MSNs) in the striatum and leads to neurodegeneration to other regions of brain, including the cortex, hippocampus, hypothalamus, and brain stem in a progressive manner [7–9]. The epidemiology of HD suggests that the disease occurs worldwide, but its prevalence varies depending upon genetic diversity and geographical regions [9, 10]. The rate of incidence of HD is considerably higher in the Caucasian population than the Asian population. While an estimate shows the prevalence and increasing trend of HD in Western Europe, Australia, North America, and the United Kingdom, India represents a large number of total HD cases in Asia [11, 12]. Single nucleotide polymorphism (SNP) at the* HTT* locus in association with the genetic diversity, lifestyle, food, and environmental factors is presumed to be the reasons for the variations in the frequency of HD among the human population [13]. HD has been characterised by choreiform movements, dystonia, cognitive deficits, and psychiatric problems [14]. These symptoms have been accompanied by neurodegeneration along with an abnormal level of neurotransmitters, microglial activation, reactive astrogliosis, and impaired neurogenesis [15]. Recently, HD patients have also been characterised with different types of behavioural, motor, and aggressive symptoms [16, 17].

All the abovementioned problems aggravate the development of HD and contribute to gradual deterioration of the physical abilities and mental processes. Importantly, people with HD have problems in taking care of their daily routine, such as food consumption, due to difficulty in swallowing (dysphagia), which may occur during the later stages of the disease. Further, abnormalities in energy metabolism caused by mitochondrial dysfunctions add to severity of the disease. The loss of muscle function in the mandibular regions, pharynx, and oesophagus could lead to disorders like bruxism (grinding the teeth), failure to intake of food and choking, which could ultimately lead to death [16, 18]. Currently, there are no available treatments that can delay the onset or arrest the progression of the disease, while the focus of medical care is limited to merely managing the neurological symptoms of HD. This is mainly due to lack of knowledge about the underlying biology of the disease.

Of the few therapeutic options available for the treatment of HD, tetrabenazine has been an approved drug by the Food and Drug Administration (FDA) for minimising the clinical symptoms of involuntary movements [19–23]. Other treatment strategies for HD include administration of antipsychotic and antidepressant drugs [21, 22]. Antipsychotic drugs like haloperidol [23], fluphenazine [24], clonazepam [25], amantadine [26], and levetiracetam [27] might help in controlling panic attacks, aggression, and choreiform movements, while antidepressants like fluoxetine [28], sertraline [29, 30], nortriptyline [31], and lithium [32] are used to stabilize depression, anxiety associated mood swings, and negative thoughts. In addition, riluzole is frequently used as a neuroprotective drug to control glutamatergic neurotransmission in HD [33, 34]. Deep brain stimulation (DBS) has been proposed as a technique to manage chorea and other motor symptoms like dystonia and cognitive deficits in HD [35]. Alternatively, using recombinant antibody fragments to neutralise the HTT aggregates [36] and stem cell transplantation [37] have also been tried with limited success. In addition, adapting to the aforementioned treatment strategies warrants a very careful approach and highly vigilant individuals to carry out the procedures, as most of these strategies and routes of administration have side effects including nausea, fatigue, abnormal neuroexcitability, and tissue disruption [38] that could exacerbate the severity of HD symptoms [39]. Thus, developing novel and noninvasive therapeutic strategies that are efficient but have no or minimal side effects are important for the successful treatment of HD and will have a promising therapeutic appeal. Meanwhile, the benefits of physical activities, an enriched environment with the aid of dietary supplements, and palliative care therapies have been considered in conjunction with the drug treatment as noninvasive and relatively affordable management strategies for HD.

## 2. Detrimental Roles of Oxidative Stress in Huntington's Disease

There have been many hypotheses proposed for the manifestation of neurodegeneration associated clinical symptoms in HD. Among them, polyQ expansion associated oxidative stress that leads to caspase mediated neuronal cell death is considered as a potential cause of neuropathological changes in HD [9]. Free radicals are highly reactive molecules that feature unpaired electrons on their valence orbital with the ability to render various molecular and cellular vulnerabilities due to their unstable reactive nature [40, 41]. The most common biologically relevant free radicals are superoxide (O^−^), hydroxyl (OH^−^), and nitric monoxide (NO) species and are referred to as reactive oxygen species (ROS) and reactive nitrogen species (RNS) [41, 42]. Free radicals are provoked in cells by enzymatic and nonenzymatic mechanisms through abnormal metabolic, genetic, and cell cycle events that occur as a consequence of electromagnetic radiation, ageing, infections, immunological alteration, intoxication, abnormal diet, malnutrition, and deficiency in vitamins and trace elements [43]. Besides, defects or mutations in the free radicals scavenging metabolic enzymes such as glutathione peroxidases, nitric oxide synthase, peroxiredoxins, and superoxide dismutases are responsible for the accumulation of free radicals [44, 45]. The expressions of these detoxifying enzymes are controlled by the Nrf2-ARE complex pathway [46]. In general, highly regulated free radicals generated in the body can be of potential use in development and maintenance of the tissues associated with improvement of the longevity of organisms. As per the free radical theory of ageing, the abnormally fabricated free radicals are harmful to the normal structure and functioning of cells and tissues [46–48]. In order to combat the negative impact of free radicals, cells deploy defense mechanisms such as free radical scavenging activity by antioxidants, as a normal physiological process [49]. The imbalance between the production of free radicals and the ability of cells to counteract or detoxify free radicals can eventually lead to DNA oxidation, protein nitration, and lipid peroxidation, culminating in cellular oxidative stress [50, 51]. Prolonged oxidative stress and failure in defense mechanisms could ultimately result in ageing related chronic diseases such as atherosclerosis [52], cancer [53], diabetes [54], rheumatoid arthritis [55], ischemic stroke [56], cardiovascular diseases [57], chronic inflammation [58], and neurodegenerative diseases including Alzheimer's disease (AD) [59] and Parkinson's disease (PD) [60], in addition to HD [61, 62].

The brain is highly susceptible to free radical mediated oxidative damage, which is largely due to its high metabolic rate and oxygen and energy consumption under the protective isolation by the blood brain barrier from the circulation [62]. ROS target neuronal cells by promoting formation of DNA-protein cross-linked harmful adducts through oxidation of both the backbone and the side chain of the protein and DNA molecules [62, 63]. Various indices of free radical mediated damage have been identified as aetiologies of several neurodegenerative conditions including HD [61]. While a reciprocal relationship exists between the length of CAG repeats and the phenotype severity of HD, recent data gathered from the experiments on the HD specific embryonic stem cells (ESC) and induced pluripotent stem cell (iPSC) models indicate the deleterious effects of oxidative damage on the expansion of CAG triplets in the* HTT* gene [64–68]. The polyQ mechanisms that account for the selective neuronal loss in the brain affected by HD are multifaceted in nature [67]. Biomarkers for oxidative damage like heme oxygenase, 3-nitrotyrosine, and malondialdehyde (MDA) are found to be elevated in the striatum, cortex, and serum of human HD subjects [69–71]. The number of polyQ repeats in the HTT protein is found to be responsible for oxidative damage to the cell membrane, DNA, and enzymes responsible for the ATP production of mitochondria in HD [70, 71]. Besides, Lim et al. reported the disruption of mitochondrial Ca^2+^ homeostasis by free radicals in the striatal neurons of postmortem human HD brains [72]. Thus, free radical induced mitochondrial damage, followed by decreased ATP production, provides a strong mechanism for provoking the apoptotic pathways in HD brains [73]. The interactions between free radical induced oxidative stress, defects in mitochondrial energy metabolism, and excitotoxicity have widely been implicated in the neuropathogenicity of HD. Evidence of the role of oxidative stress in priming the pathogenesis of HD has been identified by higher plasma levels of lipid peroxidation in presymptomatic HD patients [74–76]. In addition, the mitochondrial permeability transition pore (mPTP), a nonspecific channel, is highly susceptible to the fluctuation of calcium homeostasis and oxidative stress, which in turn are considered to be major contributors to mitochondrial dysfunction in HD [77]. Store-operated calcium entry (SOCE), a process caused by lower of Ca^2+^ from the endoplasmic reticulum (ER), induces the influx of Ca^2^ from the extracellular space. The activity of SOC channels in medium spiny neurons (MSNs) was found to be high in transgenic YAC128 mice model of HD [78]. Recently, the PPAR-*γ* pathway has been linked to the induction of superoxide/ROS in HD [79]. This suggests that the oxidative damage induced molecular and cellular changes in the circulation appear to be the initiator of the early pathogenic events in HD. Taken together, oxidative stress plays a crucial role in the neuropathology of HD and is a potential target for the development of novel therapeutic interventions for the neuroprotective management of HD.

## 3. Neuroprotective Roles of Antioxidants against Oxidative Stress-Induced Complications in Neurodegenerative Disorders

Free radicals when overproduced need to be biologically scavenged or quenched by converting them into metabolically nondestructive cellular molecules. This protective mechanism called the antioxidant defense system prevents free radical mediated damage of cells, which lead to various diseases and ageing [49, 80–82]. When endogenous antioxidant defenses are inadequate to scavenge the free radicals completely, diet or drug-derived antioxidants may be particularly important in protecting against a number of human diseases [76]. Antioxidant defense mechanisms involve both enzymatic (superoxide dismutase, catalase, glutathione peroxidase, and glutathione reductase) and nonenzymatic (vitamins A, C, and E, glutathione) strategies. Other antioxidants include albumin, bilirubin, ferritin, ceruloplasmin, melatonin, uric acid, lipoic acid, mixed carotenoids, coenzyme Q10, bioflavonoids, antioxidant minerals (copper, zinc, manganese, and selenium), and the cofactors (folic acid, vitamins B1, B2, B6, and B12) [80, 81]. Specific quenching of the free radicals and chelating redox metals by antioxidants could possibly influence the gene expression profile of the tissue. The toxic metal induced lipid peroxidation and DNA fragmentation can be controlled by metal-binding proteins like ferritin, transferrin, ceruloplasmin, and others such as metallothionein [49]. Glutathione S transferases are one of the many enzymatic entities in cells and body fluids that reduce the level of ROS [82]. Protective effects of exogenously administered antioxidants have been extensively studied using experimental animal models and cell lines and have provided a strong insight into the relationship between free radicals and associated disease complications [83, 84]. The study carried out by Chanvitayapongs et al. demonstrated the antioxidant property of resveratrol, which, when combined with vitamin C and/or E, has a greater protective effect by reducing cell death in neurodegenerative diseases including AD [85]. The amyloidal beta (A*β*) induced neurotoxicity and the underlying molecular pathological mechanisms in AD are found to be inhibited by natural antioxidants such as Ginkgo biloba, flavonoids, soybean isoflavones, theanine, and nicotine in cellular models as well as transgenic animal models of AD [86]. Long-term dietary supplementation of pomegranates, figs, and dates reduced the inflammatory cytokines during ageing in APPsw/Tg2576 transgenic mouse model of AD [87]. Essa et al. identified that a diet rich in walnut helps to reduce the risk of developing PD and delay its onset due to the cumulative antioxidant and mitochondrial protective effects exerted by walnut constituents [88]. The pomegranate oil on 3-nitro propionic acid (3-NP) induced cytotoxicity in rat pheochromocytoma (PC-12) neuronal cells enhanced the levels of enzymatic and nonenzymatic antioxidants by neutralising ROS or enhancing the expression of the antioxidant genes [89]. Rezai-Zadeh et al. demonstrated that epigallocatechin-3-gallate (EGCG), the main polyphenolic constituent of green tea through its beta-secretase activity, reduced A*β* aggregation in neuron-like cells (N2a), transfected with the human “Swedish” mutant amyloid precursor protein (APP) and in primary neurons derived from Swedish mutant APP-overexpressing Tg APP^SW^ transgenic mice model of AD [90]. L-Dihydroxyphenylalanine (L-dopa) used in the treatment of PD produced free radicals during its normal metabolism and this side effect was shown to be reduced by antioxidants in order to improve the efficacy of L-dopa therapy [91]. A series of orally bioavailable antioxidants including MitoQ, MitoVitE, and Mito TEMPOL are known to bypass the biological membranes, accumulate within mitochondria, and effectively protect against mitochondrial oxidative damage and are useful in treating neurodegenerative disease like PD [92].

In relation to the effects of antioxidants on HD pathology, a number of biomolecules have been tested and characterised using preclinical animal models of HD and cell lines expressing different length of CAG repeats. Among them, transgenic models of HD, R6-lines (R6/1 and R6/2) [93, 94], knock-in YAC128 mouse model [95], and rats or mouse injected with acute and toxic quinolinic acid [96] and 3-NP [97] have extensively been validated. In various paradigms, R6/2 mice that are supplemented with creatine, vitamin C, coenzyme Q, tauroursodeoxycholic acid (TUDCA), docosahexenoic acid (DHA), and eicosapentenoic acid showed increased life span and motor performance in association with either reduced free radicals or reduced polyQ aggregates in the brain [98, 99]. Chronic administration of JM6, an inhibitor of kynurenine-3-monooxygenase extended the life span, prevented synaptic loss, and decreased microglial activation in the R6/2 transgenic mouse and drosophila models of HD [100, 101]. A recent study indicated the significance of anthocyanin-treatment on CAG repeat instability in R6/1 transgenic mouse model of HD [102]. Treatment of experimental rats with 5-diethoxyphosphoryl-5-methyl-1-pyrroline N-oxide (DEPMPO) or with N-acetylcysteine (NAC) protects against oxidative damage induced by 3-NP and therefore acts against HD [103]. Besides, fumaric acid ester, dimethylfumarate, has been shown to provide neuroprotection and to suppress the dyskinetic movements through the activation of Nrf2 pathway in knock-in YAC-128 and transgenic R6/2 models of HD [104]. Van Raamsdonk et al. demonstrated the attenuation of striatal neuroprotection by antioxidant effects of cystamine YAC-128 model [105]. Further, treatment of melatonin significantly ameliorated the increased lipid peroxidation within the striatum of brain in the 3-NP model of HD [106]. In the same line of evidence, treatment of curcumin and carvedilol in 3-NP injected rats reduced the severity of motor and cognitive impairments [107, 108]. The treatment of resveratrol, naringin, sertraline, protopanaxatriol, embelin, puerarin, and olive oil was known to protect the experimental animal models against 3-NP induced oxidative stress and neurotoxicity [109]. Neurodegeneration in the striatum was prevented by TUDCA, a hydrophilic bile acid with antioxidant properties, which ameliorated the locomotor and cognitive deficits in a 3-NP injected rat model of HD [110]. Lycopene, a carotenoid pigment and phytochemical naturally found in fruits and vegetables, has the ability to reduce oxidative stress markers and improved behaviour in a 3-NP model of HD [111]. Low levels of cystathionine-c-lyase, required for production of cysteine, have been reported in HD pathology, which is mainly responsible for glutamate excitotoxicity. N-Acetylcysteine (NAC), an antioxidant supplement rich in cysteine, normalised the glutamate level, mitochondrial dysfunction, and oxidative stress when administered to R6/1 model [112].

The ameliorative effects of s-allylcysteine, copper, curcumin, safranal, ksheerabala, quercetin, and tert-butylhydroquinone against neurotoxicity have been described in quinolinic acid (QA) induced rat model of HD [113]. Antioxidant selenium, an essential element required by glutathione peroxidase, has been reported to reduce the lipid peroxidation within the striatum of QA rat model in a dose dependent manner [114]. Metal-containing catalytic antioxidant metalloporphyrins have emerged as a novel class of potential therapeutic agents that quench ROS in an effective manner. The dietary supplementation of lipoic acid has supported the longevity and delaying the weight loss in both the R6/2 and N171-82Q transgenic lines [115]. In addition, administration of L-carnitine dramatically extended the survival, ameliorated the motor performance, and decreased the number of intranuclear polyQ aggregates in the N171-82Q mice [116]. Another potent antioxidant, *α*-tocopherol (vitamin E), along with idebenone attenuated glutamate-induced neuronal death in HD cell lines like N18-RE-105 [117]. CDDO-MA (2-cyano-N-methyl-3,12-dioxooleana-1,9 (11)-dien-28 amide) treatment significantly attenuated 3-NP-induced loss of striatal neuronal nuclear antigen (NeuN) positive neurons [118]. Grape seed phenolic extract (GSPE) is a good metal chelator that inhibited polyQ aggregation and reduced the carbonyl levels in PC-12 cells expressing 103 glutamines fused with anEGFP reporter (HTT103Q-EGFP) [119]. However, the adverse effects of prolonged intake of antioxidants and their overdosages cannot be entirely excluded. Taken together, further investigation needs to be carried out to understand the mechanism and molecular pathways behind the above-discussed biological effects of antioxidants.

Recently, the benefits of flavonoid-rich dietary supplements have clearly been recognized in improving cognition by protecting degenerating neurons, by enhancing existing neuronal function, or by stimulating neuronal regeneration [120]. While neuroprotective natures of antioxidants against free radical damage have been extensively characterised, the neuroregenerative potential of antioxidants has recently evolved due to the neural plastic roles of adult stem cells of the brain. As a result, a number of naturally occurring dietary antioxidants have been identified with properties that support neurogenesis. The role of antioxidants has also been implicated in the functional outcomes in ageing and neurodegenerative disorders and their protective role is clearly linked to such outcomes in the abovementioned studies.

In addition, the neuroprotective effects of various natural, synthetic, and endogenous cannabinoids have been demonstrated in several in vitro and in vivo neurotoxicity models [121]. Peroxynitrite is involved in METH-induced dopaminergic neurotoxicity and the neurons can be protected against METH-induced neurotoxicity and striatal dopamine depletion by use of selective antioxidants, NOS inhibitors, and peroxynitrite decomposition catalysts [122]. Supplementation of selenium and antioxidants protect against METH-induced dopaminergic toxicity and the generation of OONO^−^ in PC-12 cell line and in mouse striatum [122]. Ascorbate is present as one among the few antioxidants in extracellular fluid and is homeostatically regulated but modulated by glutamate-mediated activity [123]. Colle et al. demonstrated that metallothioneins and metallothionein-like proteins, which are isolated from mouse brain, act as neuroprotective agents by reducing oxidative stress. Probucol (PB), a phenolic lipid-lowering agent, possesses antioxidant property of scavenging free radicals and acts as a NMDA receptor antagonist, thereby promoting neuroprotection [124]. However, none of the abovementioned studies could suggest effective treatment strategies that could completely reverse the disease pathology. Effective management of degenerative diseases cannot be achieved by strategies that focus only on neuroprotection, whereas neuroregeneration through stem cell mediated adult neurogenesis needs to be promoted, in order to compensate for the functional deficits that occur due to neuronal loss.

## 4. Functional Significance and Regulation of Adult Neurogenesis

**Figure 1:**
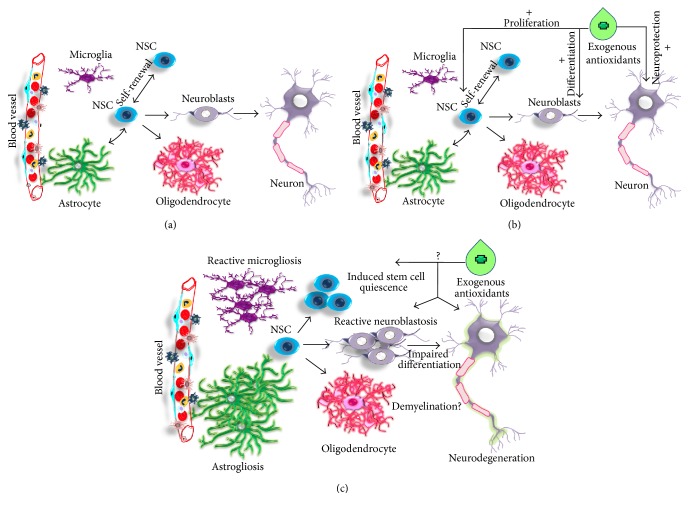
Illustration of neurogenic niche of the adult brain in various conditions. (a) In normal brain; (b) under antioxidant supplement; (c) neuropathology and neurogenesis in HD.

The adult brain retains the capacity to generate new neurons by the process called neurogenesis in specific regions of the organ [125–128]. The actively occurring neurogenesis is restricted to three defined neurogenic regions in the adult brain under normal conditions, namely, (1) the subgranular zone (SGZ) in the dentate gyrus (DG) of the hippocampus [126, 127]; (2) the subventricular zone (SVZ) of the lateral ventricles [129]; and (3) third ventricles of the hypothalamus [130]. In the hippocampal SGZ, proliferating NSCs develop into intermediate progenitors, which generate neuroblasts or immature neurons. These newly generated immature neurons migrate into the inner granule cell layer (GCL) and differentiate into new granule neurons of the hippocampus [128]. Further, these newborn neurons extend dendrites from DG towards the molecular layer (ML) and project axons that form the mossy fibber tract in the hilus region. In the SVZ, proliferating radial glia-like cells give rise to transient amplifying cells that generate migrating neuroblasts [128, 131]. Through the rostral migratory system (RMS), neuroblasts migrate towards the olfactory bulb (OB) [131]. In the OB, immature neurons differentiate into different subtypes of interneurons in granule cell layer (GCL) and glomerular layer (GLOM). Neural progenitor cells identified in the ependymal layer of the third ventricle of the adult brain migrate and differentiate into mature neurons in the hypothalamus [130] (Figure 1).

The potential roles of adult neurogenesis in various neurophysiological processes like motor functions, learning and memory process, olfaction, and the regulation of hypothalamus-pituitary-adrenal (HPA) axis have been extensively characterised [130, 132]. Adult neurogenesis has been known to be an integral component in neural plasticity, brain homeostasis, maintenance, and tissue remodelling [130]. Adult neurogenesis is a multistep process that includes stem cell proliferation, cell cycle exit, and fate determination of adult neural progenitors followed by differentiation, maturation, and integration of mature neurons into the neural circuits [130, 133]. This process has been shown to be modulated by many positive and negative factors [128, 130–134].

The ageing process, exposure to prolonged stress, abnormal levels of glucocorticoids, radiation, prolonged drug abuse, and chronic neuroinflammation are known to negatively influence adult hippocampal neurogenesis by inhibiting the proliferation and differentiation of NSCs or promoting the cell death of newborn granule cells [130, 135, 136]. This could lead to cognitive decline and loss of control of the HPA axis and may render the susceptibilities to neuropsychiatric and neurodegenerative disorders leading to cognitive impairments [137, 138]. Moreover, many of neuropsychiatric and neurodegenerative disorders are characterised with impaired adult neurogenesis. It has been shown that neurogenesis is impaired in the hippocampus of transgenic R6 mouse lines [139–141], transgenic rat model of HD [142], and knock-in YAC128 model [143]. However, in chemically induced acute neurodegenerative models and HD patients, neurogenesis is increased in the SEL and SVZ, respectively [144, 145]. The increased neurogenesis in the SVZ in combination with abnormal migration of neuroblasts in the striatum is also observed in a toxic rat model of HD [145]. Thus, the abnormal reactive neurogenesis in the striatum has been observed as a common hallmark in HD [146]. Acute neurological conditions like stroke, epilepsy, and traumatic brain injuries have also been associated with increased adult neurogenesis [147, 148]. In HD, the reactive neurogenesis in striatum has been speculated to be an attempt of stem cell mediated regeneration to overcome neuronal dysfunctions and neuronal loss. Thus, the endogenous self-regenerative measures adapted by HD brain through neurogenesis to overcome neurodegeneration highlight the possibility of exploiting promotion of antioxidant-mediated neural regeneration as a management strategy for HD.

The positive regulators of neurogenesis comprise physical activity, environmental enrichment, growth factors, and antioxidants derived from diet. It is evident that physical activity such as running promotes neurogenesis by increasing the proliferation of NSCs in the SGZ of the dentate gyrus, thereby expanding the pool of progenitor cells available for further neuronal differentiation in the hippocampus [149]. Spontaneous physical activity and task-based learning are the two important components of an enriched environment that promotes hippocampal neurogenesis [150]. Neurotrophic factors, cytokines, and growth factors regulate the adult neurogenesis by controlling proliferation, maturation, differentiation, and survival of neuronal cells. Systemic infusion of insulin growth factor-1 (IGF-I) increased the proliferation of NSCs, frequency of neuronal differentiation, and survival in the adult rat hippocampus [151]. Jin et al. demonstrated that the angiogenic protein, vascular endothelial growth factor (VEGF), stimulates the proliferation of NSCs in murine cerebral cortical cultures and in adult rat brains, thus promoting neurogenesis apart from angiogenesis [152]. Intracerebroventricular infusion of epidermal growth factor-1 (EGF-1) and fibroblast growth factor-2 (FGF-2) increased proliferation of NSCs in the SVZ of the adult rat brain [153]. Ciliary neurotrophic factor (CNTF) supported adult neurogenesis as CNTF knockout mice showed reduced neurogenesis [154].

Cotman and Berchtold reported that voluntary exercise increased the levels of brain-derived neurotrophic factor (BDNF) and other growth factors which in turn stimulated neurogenesis [155]. Nonsteroidal anti-inflammatory drugs (NSAIDs) are recognized to increase adult neurogenesis and thus aid in the process of neuroprotection [156]. Chronic treatment with various antidepressants like tranylcypromine, reboxetine, fluoxetine, and haloperidol is reported to increase neurogenesis in early adulthood and experimental models of stress [157]. It has been reported that neuronal differentiation and survival are associated with TGF-beta signaling and thus TGF-beta signaling remains as a crucial regulator of adult neurogenesis [15, 142, 158]. Moreover, it has also been reported that adult neurogenesis is positively regulated by diet and it could prevent cognitive decline during ageing, as well as to counteract the effect of stress and depression [159]. It was shown recently that a reduction in calorie intake increases hippocampal neurogenesis in adult rodents and that this effect is partly mediated by BDNF [160]. Taken together, the decline in brain plasticity and mental process can possibly be reestablished by the activation of NSCs that have the ability to self-renew and develop into neurons or glial cells. As no specific treatments are available as a cure to HD, neuronal stem cell (NSC) mediated neurogenesis provides a regenerative strategy to replace the neuronal loss and neural plasticity including motor impairments, cognitive functions, and mood that are affected in HD.

## 5. Role of Oxidative Stress on the Regulation of Adult Neurogenesis

**Figure 2:**
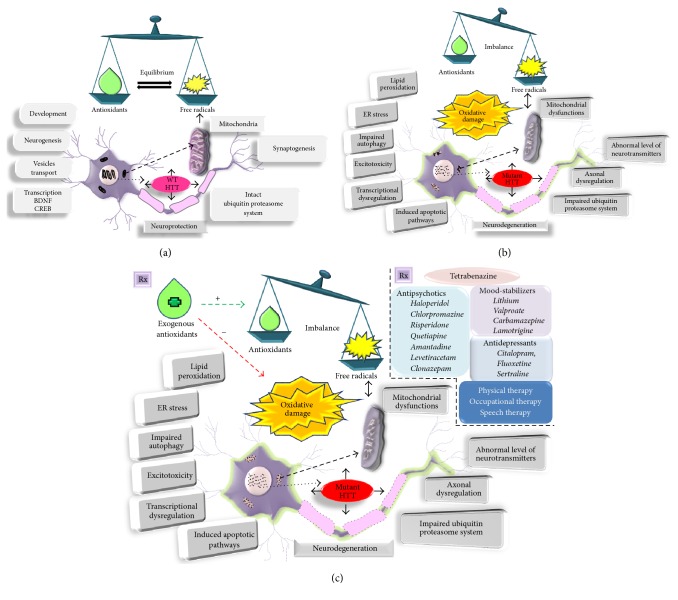
Graphical representation for the possible roles of free radicals and antioxidants on neuronal functions in control and HD conditions. (a) A balance is maintained between the amount of free radicals generated and the antioxidant defense mechanism which aid in normal physiological functions of the neuron in normal brain. (b) In HD brain, the free radicals generated are higher and the antioxidant defense mechanism is impaired, resulting in oxidative damage. (c) Various therapeutic options to overcome the disruption caused by oxidative stress on cellular functions of neuron in HD.

The functional impairments of NSC, particularly neurogenesis, represent an increasingly prominent contributor to multiple CNS diseases and the process is highly altered by elevated levels of oxidative stress. Oxidative stress caused by increased ROS has been considered to affect neurogenesis and cognitive functions [161]. Acute exposure of NSCs to ketamine leads to increased cell proliferation whereas the chronic incubation results in cellular damage via altered mitochondria pathways and induces cellular apoptosis [162]. Superoxide dismutases (SODs) scavenge the superoxide radicals by converting them to hydrogen peroxide and oxygen molecule thus acting as first-line defense to protect cells [163]. The SOD deficient mouse model showed reduction in the generation of new neurons in the hippocampus upon cranial irradiation [164]. Cranial irradiation, an effective treatment for brain tumors, leads to persistent elevation of oxidative stress and suppression of hippocampal neurogenesis [165]. Increased oxidative stress following irradiation is expected to play a major role in the suppression of hippocampal neurogenesis and the associated cognitive deficits. Walton et al. suggested that the production of ROS is a part of the routine maintenance of physiological neurogenesis, but chronic oxidative stress may play a role in loss of function in ageing and progressive CNS diseases [165]. Accelerated age-dependent decline in adult neurogenesis is a consequence of oxidative stress. Conditional deletion of the clock gene* Bmal1* (Bmal1^−/−^) in mice accelerated ageing, neurodegeneration, and cognitive deficits through oxidative damage [166]. Moreover, oxidative stress promotes aneuploidy and formation of neurofibrillary tangles in the neurogenic regions of the brain, contributing to neurodegeneration in AD. Sirtuin protein family members (e.g., Sirt1, Sirt2) are considered to be important in determining the redox state in NSCs and also provide potential targets for modulating adult neurogenesis [167]. Taken together, increased levels of oxidative stress by high accumulation of ROS have negative effect on adult neurogenesis during ageing, neuroinflammation, and neurodegeneration [168]. Thus, targeting oxidative stress represent a novel way to regulate adult neurogenesis. This in turn will help in decreasing the pathogenesis of neurocognitive disorders including HD by promoting neurogenesis in order to compensate the neuronal loss, which could pave a path for supporting the cognitive functions (Figure 2).

## 6. Supportive Role of Antioxidants in Promoting and Regulating Adult Brain Neurogenesis

The cognitive health of an organism is maintained by the capacity of hippocampal neurogenesis. Recently, benefits of antioxidants have emerged as a potent strategy to support the cognitive function through the regulation of adult neurogenesis. Consumption of potent antioxidants, for example, melatonin and polyunsaturated fatty acids, has a significant effect in lowering the decline of neurogenesis and attenuating the impairment of cognitive function [169–171]. Impairment of hippocampal neurogenesis in rat models of chronic alcoholism by elevation of oxidative stress can be reversed by treating with ebselen, a drug with antioxidant property [152]. Curcumin has been shown to increase adult neurogenesis in the hippocampus of adult rodents. An antioxidant-fortified food in an enrichment plan affected the survival of new neurons in the aged canine brain and is associated with improvement in cognitive performance [170]. Administration of flavonoids like 3′-methylated epicatechin and 4′-methylated epicatechin to animal models improved cognitive performance by promoting neurogenesis [171]. Qu et al. demonstrated that* Rhodiola crenulata* extract (RCE), containing a potent antioxidant salidroside, promotes neurogenesis in the hippocampus of intracerebroventricular injected streptozotocin model of AD [172]. Polyphenols are abundant micronutrients present in plant-derived foods, fruits, and beverages such as tea, red wine, cocoa, and coffee and also act as powerful antioxidants [173]. In rats, polyphenols increased hippocampal plasticity and improved learning and memory performance, while protecting neurons against injury induced by neurotoxins suppress neuroinflammation and the potential to promote cognitive function. In general, dietary polyphenols seem to exert positive effects on anxiety and depression via regulation of adult hippocampal neurogenesis [174]. Flavonoids protect the brain in many ways through enhancement of existing neuronal function or by stimulating neuronal regeneration [175]. Polyphenol-rich Ginkgo biloba extracts and other flavanoids have been shown to protect hippocampal neurons from oxidative stress, nitric oxide, and beta-amyloid-induced neurotoxicity [176]. An et al. reported that the supplementation of flavanoids (XBXT-2) in rats subjected to chronic stress shows increased neurogenesis and increase in BDNF levels [177]. Different polyphenols are shown to exert their effects on adult hippocampal neurogenesis via different mechanisms of action, such as by activating the MAP kinase pathway or stimulating the expression and release of neurotrophic factors [178]. Cocoa powder and chocolate contain a large percentage of flavonoids, mainly epicatechin that interacts through signaling cascade proteins and lipid kinases thereby inhibiting neuronal death by apoptosis induced by oxygen radicals and promoting neuronal survival and synaptic plasticity [179]. In addition, flavonoids preserve cognitive abilities in rats during ageing and lower the risk of AD stress and stroke in humans [179]. Thus, neurogenic properties of antioxidants have great significance in therapeutic interventions for brain diseases. However, reports on the effect of antioxidants in regulating adult neurogenesis in HD are limited. While R6/2, R6/1, YAC128 mice, and TgHD rats have been characterised with induced NSCs quiescence and impaired neurogenesis, the elevated level of neuroinflammation related factors like TGF-beta appears to inhibit the regenerative ability of the HD brain [15]. Free radicals involved oxidative damage observed in HD might also act synergistically with neuroinflammation to impede the proneurogenic signals in HD. Indeed, combinations of antioxidant therapy along with physical exercise may exert beneficial effect to promote neuroregeneration in HD.

HD is accompanied by both cognitive and motor defect, which is caused by progressive loss of striatal neurons. Increased neuronal cell death has also been described in the cortex and the hippocampus of HD brains in addition to the striatum [9]. Recently, adult neurogenesis has been identified in regions other than hippocampus and SVZ-OB such as amygdala and hypothalamus which are responsible for fear, memory, and the regulation of neuroendocrine functions, respectively [180, 181]. Interestingly, it has also been reported that neurogenesis occurs in the cortex of the adult monkey [181, 182] and normal rat [183], suggesting that neurogenesis of adult brain is widespread. Induction of adult neurogenesis in response to many CNS trauma and neurological diseases has been reported by several studies in the past decade [184, 185]. It has been demonstrated that ischemia is a well-known factor to contribute to reactive neurogenesis in the cortex [147, 148, 186, 187]. The abnormal cortical neurogenesis has also been reported in multiple sclerosis [188] and ALS [189]. Considering these facts, it can be speculated that regeneration of damaged brain through cortical neurogenesis can occur in the brains of HD subjects. However, reports on adult cortical neurogenesis remains a subject of ongoing debate [190]. Hence, these findings need to be reconfirmed and validated with better experimental models. Future studies should focus on confirming the occurrence of neurogenesis in the cortex of damaged adult brains, since cortical neurogenesis is important for compensating the loss of motor functions in HD cases.

While a new line of research focusing on cortical neurogenesis is necessary to implicate its role in brain regeneration, reactive neurogenesis in the striatum of both normal and pathogenic adult brain including HD subjects has recently been well established [143, 145, 191]. As the striatum plays an important role in the planning and modulation of movement, it raises a question whether striatal neurogenesis is required for compensating loss of motor tasks in HD. Abnormal cell proliferation and reactive neuroblastosis in the striatum have been observed in several cases of HD brains [146] as a mechanism to replenish the loss of neurons in the striatum. However, in many such cases the migrating and resident neuroblasts undergo apoptosis before maturing into neurons in the striatum. Hence, survival of neurons does not occur in the striatum of HD [143, 191, 192]. As a part of compensatory mechanism against QA striatal lesion-induced neuronal loss, the brain promotes neurogenesis in the SVZ, from where neuroblasts migrate to the damaged areas of the striatum [144]. Ernst and Frisén demonstrated the presence of neuroblasts in the striatum of the human brain using doublecortin (DCX) and polysialylated neuronal cell adhesion molecule (PSA-NCAM) immunostainings and confirmed the occurrence of neurogenesis in the striatum [191, 192]. However, they did not observe the survival of newborn neurons in the normal striatum as well as in HD cases, confirming the premature depletion of neuroblasts before their integration into the striatal tissue. The failure of neuronal differentiation in the striatum and reactive striatal neuroblastosis has been recapitulated in the rodent models of HD [143, 144, 146]. Taken together, it can be hypothesized that the migrating SVZ born neuroblasts and/or neuroblasts generated by the striatum might provide the neurophysiological support to the striatum to overcome the motor deficits to certain extent. In this respect, it will be very interesting to see if antioxidants can contribute to the terminal differentiation, integration, and survival of reactive neuroblasts in the degenerated striatum of HD. It is possible that antioxidants may provide substratum and trophic support in addition to mitigating ROS generated by striatal gliosis and neurodegeneration in HD.

## 7. Conclusion

HD is a progressive neurodegenerative disease that has been refractory to treatment. Despite the enormous research focus on HD, no valid treatment that can alleviate the symptoms of HD has been developed. This could be attributed to the complex nature of the disease and lack of evidence on a precise molecular target for therapeutic intervention. Generation of free radicals leading to oxidative stress (OS) damage contributes to neuronal loss in HD and the oxidative stress could be reduced by supplementation of natural antioxidants. Adult neurogenesis can act as an important tool for regenerative therapy of HD brains as it contributes to the cognitive functions of the adult brain. Neurogenesis has been shown to be upregulated by numerous antioxidants. Impaired hippocampal neurogenesis and reactive striatal neurogenesis have been the characteristics of HD brains. Naturally occurring antioxidants might therefore provide neurotropic as wells as proneurogenic and neuroprotective support for the HD brain, in order to overcome the motor and cognitive impairments. However, the complete relationship between oxidative stress and neuroregeneration, and the molecular mechanism by which antioxidants support the process of adult neurogenesis by triggering various signaling cascades, needs further diligent investigation.

## Abbreviations

HD: Huntington's disease
CAG: Cytosine-Adenine-Guanine
PolyQ: Polyglutamine
ROS: Reactive oxygen species
RNS: Reactive nitrogen species
MSN: Medium spiny neurons
DG: Dentate gyrus
SGZ: Subgranular zone
SVZ: Subventricular zone
OB: Olfactory bulb
SNP: Single nucleotide polymorphism
FDA: Food and Drug Administration
Nrf2: Nuclear factor (erythroid-derived 2)-like 2
AD: Alzheimer's disease
PD: Parkinson's disease
ESC: Embryonic stem cells
IPSC: Induced pluripotent stem cells
MDA: Malondialdehyde
HO: Heme oxygenase
ATP: Adenosine triphosphate
MTA: Mitochondria-targeted antioxidant
mHTT: Mutant huntingtin protein
DHA: Docosahexenoic acid
TUDCA: Tauroursodeoxycholic acid
NSC: Neuronal stem cell
GCL: Granule cell layer
RMS: Rostral migratory system
GLOM: Glomerular layer
HPA: Hypothalamus-pituitary-adrenal
IGF-I: Insulin growth factor-1
VEGF: Vascular endothelial growth factor
EGF-1: Epidermal growth factor-1
FGF-2: Fibroblast growth factor-2
CNTF: Ciliary neurotrophic factor
BDNF: Brain-derived neurotrophic factor
NSAIDs: Nonsteroidal anti-inflammatory drugs
CNS: Central nervous system
SODs: Superoxide dismutases
RCE: * Rhodiola crenulata* extract
APP: Amyloid precursor protein
A*β*: Amyloid beta.
